# Experiences of Iranian Nurses on the Facilitators of Pain Management in Children: A Qualitative Study

**DOI:** 10.1155/2016/3594240

**Published:** 2016-03-30

**Authors:** Parvin Aziznejadroshan, Fatemeh Alhani, Eesa Mohammadi

**Affiliations:** Department of Nursing, Faculty of Medical Sciences, Tarbiat Modares University, Jalal Al-Ahmad, P.O. Box 14115-331, Tehran, Iran

## Abstract

*Background*. Despite decades of research and the availability of effective analgesic approaches, many children continue to experience moderate-to-severe pain after hospitalization. Greater research efforts are needed to identify the factors that facilitate effective pain management. The aim of this study was to explore the perceptions of Iranian nurses on facilitators of pain management in children.* Materials and Methods*. This qualitative study collected the data profoundly through unstructured interviews with 19 nurses in Amirkola Children's Hospital in Babol and Children's Medical Center in Tehran, during 2013-2014. Purposeful sampling and analysis of the data were conducted using conventional qualitative content analysis.* Results*. Four themes were extracted through data analysis: mother and child participation in diagnosis and pain relief, the timely presence of medical staff and parents, proper communication, and training and supportive role of nurses.* Conclusion*. Mother and child participation in the report and diagnosis of pain and nonpharmacological interventions for pain by the mother, the timely presence of medical team at the patient's bedside, and proper interaction along with the training and supportive role of a nurse enhanced the optimal pain management in hospitalized children.

## 1. Introduction

Pain is one of the most common reasons why people visit doctors or health team members and, in many clinical conditions, pain is a major symptom. In children, pain reduces the quality of their life, so pain preventive measures in all areas need to be taken [1]. Despite improved pain management procedures during the past forty years, pain remains a global problem for hospitalized patients [2]. The prevalence of pain in hospitalized children was 86% and almost 40% had moderate-to-severe pain [3]. Pain can have physical, emotional, and psychological impact on school attendance and isolation of these children from their friends [4]. Inadequate management of pain can cause ineffective and increased duration of hospitalization [5]. Pain in hospitalized children is often inadequately treated; pediatric nurses have experienced these shortcomings more than other health care team members [6]. Pain management is one of the most important indicators in measuring the quality of care [7]. Pain management and assessment are an integral part of nursing care [8]. Effective pain management can minimize the negative physiological and behavioral effects in children and shorten the days connected to ventilator and oxygen therapy, weight gain, improvement, and reduction in the duration of hospitalization [9]. Despite improved review and management of pain, pediatric pain relief in many countries is incomplete [10, 11]. The need to enhance pain management is well documented [12]. Effective pain management requires accurate knowledge, attitude, and skills of nurses [13]. Twycross believed that teaching the nurses, improving manpower, equipment availability for distraction, and appropriate tools to assess pain can make better management of pain in children [14]. For greater accuracy in assessing pain in children and the effectiveness of its management, it is important that parents participate in the process of assessing pain to improve health care [15]. Parents needing communication and emotional support during the hospitalization of their children in care unit has become global [16]. The American Academy of Pediatrics has emphasized the parental presence during a painful procedure as a necessity protocol [17].

The factors affecting children's pain management are influenced by cooperation (nurses, doctors, parents, and children), child (behavior, diagnosis, and age), organization (lack of routine instructions for pain relief, lack of time, and lack of pain clinics), and nurses (experience, knowledge, and attitude) [18].

Despite the fact that a variety of clinical studies on all aspects of pain have been conducted in many countries, factors affecting pain management, especially facilitating factors, have not been fully identified [19]. This qualitative study aimed to assess the perception of Iranian nurses on the facilitators of pain management in pediatric units and centers.

*Context*. There is only a guideline on analgesia (from the Ministry of Health and Medical Education) in pediatric ward, according to which physicians act. Based on this protocol, oral analgesics and/or suppository of acetaminophen are initially used for pain relief. In the next stage, injectable acetaminophen (Apotel) and pyridamole are administered. In case of untreated pain, the third-stage analgesics like pethidine and morphine or methadone pills for severe pediatric pain are prescribed. Psychological counseling is the last pain relieving stage in this guideline. There are no specific protocols or guidelines on nonpharmacological pain interventions. There is also no pain control team or nursing pain committee in hospitals.

## 2. Materials and Methods

This qualitative study was carried out aiming at understanding Iranian nurses' perceptions and facilitators of pain management in pediatric wards, using conventional content analysis. This method is usually used for describing a phenomenon where there are limited numbers of relevant theories or researches [20]. The participants consisted of 19 nurses (16 nurses and 3 head nurses) working in different units of two teaching pediatric hospitals, namely, Amirkola Children's Hospital in Babol and Children's Medical Center in Tehran (Table 1). To initiate interviews, purposive sampling was used. The inclusion criteria include the following: at least three years of working experience in different pediatric wards, enough experience in pain management, willingness to participate, and good verbal ability for transferring experiences. Before the interview, participants were assured of the confidentiality and voluntary nature of the study. Furthermore, all nurses were required to give written informed consent.

### 2.1. Data Collection

Simultaneous data collection and analysis were performed to develop the themes related to the nurses' perceptions of facilitators of pain management. Data collection process was continued until data saturation was reached in the last three interviews. After obtaining nurses' written informed consent, interview appointments were arranged. The unstructured individual interviews were carried out in a quiet room in the hospitals. The interviews, lasting from 60 to 120 minutes, were recorded with the permission of the interviewees. Interviews began by asking questions such as the following: “Please, describe your routine work shift.” Questions were concentrated on pain management cases; if not, the next question was asked: “Have you ever encountered pediatric pain in your work shifts?” The interviewer then asked, “Please, tell us your experience with regard to pediatric pain.” Afterwards, more explanatory and in-depth questions were asked (“Please, describe more.” “What do you mean?”).

### 2.2. Data Analysis

Data collection and analysis were done at the same time. This study used conventional content analysis. Graneheim and Lundman have proposed the following steps to use content analysis with qualitative data: (1) transcribing the whole interview immediately after it is done, (2) reading the whole transcribed text to gain a general knowledge of its content, (3) determining the meaning units and initial codes, (4) categorizing similar initial codes in more comprehensive groups, and (5) determining the main theme per category [20] (Table 2). Analysis procedure was as follows: the recorded interviews were immediately transcribed in Word. Afterwards, the transcribed interviews were reviewed many times to obtain a general understanding of the nurses' opinions. The initial codes were elicited and categorized by similarities and differences. To ensure data accuracy and reliability, four criteria, namely, credibility, confirmability, dependability, and transferability, were used according to Lincoln and Guba's criteria [21]. To increase data credibility in this study, prolonged engagement and participant review strategies were employed. The prolonged engagement with participants (for two years) was performed by a researcher through long-term on-site observation of pain management by nurses. After coding process, the transcribed interviews were returned to the participants to ensure the accuracy of codes and interpretations. To control the conformability of data, the opinions of two research team members, who were experienced in qualitative studies, were used. Discussions on codes and categories disagreed on were continued by research team members until the clarity of subject and consensus was achieved. To check the dependability of data, the obtained categories were given to three nurses, not the study participants, to confirm data fit. To check data transferability, sampling was done with maximum variance of nurses (Table 1).

### 2.3. Ethical Considerations

The samples were informed about the purpose and method of the study. They were assured that they could withdraw from the study at any time without being penalized. Finally, written informed consent was obtained from the nurses who willingly declared their participation in the study.

## 3. Results

Four themes for facilitating pain management were identified: mother and child participation in diagnosis and pain relief, the timely presence of medical staff and parents, proper communication, and training and supportive role of nurses.

### 3.1. Mother and Child Participation in Diagnosis and Pain Relief

According to mother's report to the nurse about the side effects after injection, mother helps the nurse perform nonpharmacological pain relief interventions, assesses immature and unconscious children's pain and reports pain to the nurse, reminds the nurse of child repeated pain, and also notifies the nurse in case the nurse herself forgets the pain because of shortage of nurses or crowded wards.

*Due to lack of work force, I cannot be with the patient, and I have to ask mothers to perform the non-pharmacologic interventions so I can perform my other patients' needs. (Nurse 9) *

One of the facilitating points of mothers in pain management is their cooperation and assistance in examining the pain. Mothers have an active role in examining the pain in young and unconscious children, and pain is reported by the mother to nurse.

*When a child has pain, the mother reports to me about the location of the pain, when it started, having had a history and a series of information that a mother can provide to help me. (Nurse 3)*

*If a child is not old enough to answer or not so alert, then I ask the mother. (Nurse 16)*

Nurses pay attention to the mature and conscious child's information and participation in reporting the location and severity of pain.

*Alert and mature child can help me find the pain such as what the pain is like, quality of pain, where the pain comes from and when it started. I ask these questions from the child. (Nurse 10)*

### 3.2. Timely Presence of Medical Staff and Parents

Nurses mentioned the timely presence of the medical team at the patients' bedside as an accelerator factor in pain relief, emotional support of the family, and better understanding of the patient and a precipitating factor in the patients' complaints of pain.

*If a mother says my child is in pain, I immediately go by the child's beside to see the severity of pain, when the pain is severe, I resort to medication. (Nurse 13)*

Easy access to doctor accelerates pain relief.

*Because this is a training hospital, doctors are always available for pain, and I call them immediately for pain relief. (Nurse 2)*

Nurses report that meeting relatives and friends can be an accelerator for pain relief and boosting the morale of the children for pain, reduce stress and calm the child, and accelerate the consumption of housing and nonpharmacological interventions to pain.

*Procedures such as venipuncture can be better performed beside a companion, so I call the security for patient's companion to calm down the child. (Nurse 19)*

### 3.3. Proper Communication

One of the obtained themes was proper communication between nurses and patients, companions, and doctors. Friendly relationship between nurses and patients, companions, and physicians is the success factor in pain management and the use of nonpharmacological interventions.

*When I established a good relationship with the patient, they trust me about the non-pharmacological methods of pain relief. (Nurse 15)*

*Sometimes residents are busy and when the patient is in pain, I have to call the hospital attendants since we have a good working relationship. (Nurse 7)*

### 3.4. Training and Supportive Role of Nurses

Nurses have training and supportive role by explaining the side effects of painkillers and teaching the nonpharmacological interventions to the mother before painful procedures to endure pain better and also to give her peace of mind.

*When mothers insist on analgesic injection, I explain the side effects of pethidine for them. (Nurse 6)*

*When infant are fidgety, I teach the mother how to hug the baby and give the baby a proper position. (Nurse 9)*

A participant said the following about the supporting role of nurses for mother and child:

*When a child with a leukemia diagnosis is in pain, I go and cuddle the patient to calm him/her. (Nurse 17)*

Explanation of a nurse can give comfort to the mother.

*In the Emergency Department, because of pain, children are restless, parents get fidgety too by watching their child in pain, then I explain the situation to parents and calm them down. (Nurse 5)*

## 4. Discussion

For the purpose of this study, the perceived meaning of the facilitators of pain management included the following: mother and child participation in diagnosis and pain relief, the timely presence of medical staff and parents, proper communication, and training and supportive role of nurses.

The outcomes of this study suggested that mother and child participation in diagnosis and pain relief is one of the facilitating factors in pain management in pediatric wards. The role of the nurse is to encourage mother to participate in nonpharmacological interventions and cooperate with the nurse in daily physical care. This finding is consistent with previous studies on the cooperation of parents in nonpharmacological interventions of pain relief in helping nurses because they are accessible and familiar with the child's daily activities [14, 22]. Pölkki et al. also mentioned that “the child and parents' cooperation is a factor in promoting the non-pharmacological methods of pain control” [23]. Unlike these studies, Simons et al. found that parents' involvement in childcare is superficial and they have passive role and convey feelings of frustration to child [24].

The results also showed that mothers have active participation in pain assessment of young and nonconscious children, and pain is reported by the mother to nurse. These results are consistent with Lim et al.; in their study, parents regularly observed and evaluated children in terms of pain, its location, and severity and have active participation in pain monitoring, although they do not use pain assessment tools [25]. The study of Twycross reported that the nurses also noted that parents should inform nurses when their child is in pain; most parents recognize the pain and they know better drug effects [14]. Parents' participation facilitates the shortage of nurses due to overload [24]. But the participation of children in the control of pain is limited. Nurse pays attention to information and participation by mature and conscious children in considering the report of pain. Pölkki et al. reported similar findings that the parents' cooperation and participation in pain management and the child's age and his ability to cooperate are factors promoting the use of nonpharmacological pain management strategies [23]. Children can even offer help to nurses in times of pain [14]. Nurses who are under more pressure at work may face significant challenges and the participation of children can facilitate the decision-making for taking care of their pain [26].

The results of a recent study have shown that the highlighted presence of medical staff and parents is another factor facilitating the pain management in pediatric wards. But they are in contrast to Panahi et al.'s study which reported that identifying the lack of timely presence of nurse by patient is the source of conflict between doctors and nurses [27]. The findings also demonstrated that nurses notified the presence of patients' companions as the facilitators in helping in relieving the pain and also in nonpharmacological interventions. This finding is consistent with a study conducted by Pölkki et al., in which parents have been identified as a strategy for pain relief [23]. Parental presence was able to provide emotional support [25], although the outcome in Namnabati et al.'s study was inconsistent because of the negative attitude towards the mother's presence during procedures [28]. The findings also indicated the warm and friendly relationship and cooperation between the nurses and the doctors. Although the treatment of chronic pain is challenging, in the study of Butow and Sharpe and Ishikawa et al., good communication between health providers and patients improves the outcomes in pain management [29, 30]. The results of the study also showed that there was warm and friendly relationship between patients and nurses. Study findings indicated that intimate relationship between nurses and patients and their companions was the success factor in pain control and the use of nonpharmacological interventions.

In fact, the interaction between nurse and patient can be used as criteria of efficient and effective care [31]. But Simons' findings showed that poor communication between parents and nurses and nurses' lack of knowledge regarding pain management are the barriers in the way of effective pain management [32]. These barriers need to improve children pain management through better training and wide approach to communication with parents. These results can be a guide for nurses to understand that cordial relationship with children and parents, in addition to the professional relationship, is an important factor affecting children's pain management.

The findings of a recent study have shown that nurses perform their training role by describing the side effects of analgesics and teaching the nonpharmacological interventions to parents. The results indicated that providing information to parents has been effective in reducing anxiety and feelings of unreality, and lack of sufficient information was identified as the main barrier to the parents' participation in the health care of a hospitalized child [25, 33]. Studies illustrated that parents need information and tips for pain management and pain relief with nonpharmacological interventions for children to mitigate concerns about the children's condition and the effectiveness of pain relief methods [16, 22, 33]. Lovell et al.'s study demonstrated the effectiveness of teaching the patient about pain relief and for optimal results the standard method should be used in all hospitals [34]. Nurses need to be trained on nonpharmacological methods of pain relief. Educational intervention by nurse guides parents in the use of nonpharmacological methods of pain relief in children. Continuing education of pain management prepares nurses regarding the knowledge to improve clinical care [35, 36].

The results showed that the supportive role of nurses is done through fondling and touching the child's face or giving an explanation to the children and families. Studies showed that nurses used emotional support such as reassurance and touching strategies and sometimes words like relax, please, or try to endure the pain to reassure the child. Moreover, parents try to search for information about procedures to prepare their children, including tips/knowledge of nurses [25, 37]. Research studies showed that parental support is an important factor to promote pediatric pain management. Parents feel their supportive network and emotional support of nurses can help them in the care process of children in hospital [25, 38].

## 5. Limitations of the Study

Regarding nursing care plan in Iran, childcare is most often done by female nurses; therefore, noninvolvement of male nurses may drop some aspects of pain management that relate to nurse's unknown gender. The involvement of male nurses in future studies is recommended to explain the facilitators of pain management in pediatric wards.

## 6. Conclusion

Mother and child participation in the report and diagnosis of pain and nonpharmacological interventions for pain by the mother, the timely presence of medical team at the patient's bedside, and proper interaction along with the training and supportive role of nurse enhanced the optimal pain management in hospitalized children.

## Figures and Tables

**Table 1:** Participants' demographic characteristics.

Participants	Age (years)	Work experience (years)	Educational attainment	Medical units	Marital status
16 nurses 3 head nurses	29–49	5–24	Bachelor's degree: 15 Master's degree: 4	Internal (6) Surgery (5) Intensive care (3) Emergency (5)	Single: 5 Married: 14

**Table 2:** The process of developing the “timely presence of medical team and parents” theme.

Meaning unit	Subcategory	Category	Main theme
“Procedures such as vein puncture can be better performed with the presence of a companion, so I call the security to ask for the child's companion to calm down the child.”	Visits to boost child's morale for pain	The presence of companions accelerates pain relief	Timely presence of medical team and parents
“Sometimes I do not interfere and let the mother do it, if the child is calm, he/she can easily accept non-pharmacologic interventions.”	Parents' presence accelerates the nonpharmaceutical interventions		
“If mother said my child is in pain, I immediately go the child's bedside to see the severity of pain, when the pain is severe, I resort to medication.”	The continuous presence of nurses by patient accelerates pain relief	The presence of medical team accelerates pain relief	
“Because this is a training hospital, doctors are always available for pain, and I call them immediately to relieve the pain.”	Ease *of availability of physicians* accelerates pain relief		

## Additional Points

1. Mother and child help the nurses in diagnosing and reporting pain and nonpharmacological interventions.
2. The timely presence of the medical team at the bedside of the children was an emotional support and also a facilitating factor to deal with patients' complaints regarding pain.
3. The presence of parents reduced the stress and taking painkillers and helped the nonpharmacological interventions.
4. Availability of physicians as facilitators relieves children's pain.
5. The good rapport between nurses and patients, caregivers, and physicians causes better success in the pain management.
6. The explanations given by the nurse before the painful procedures cause children to better tolerate the pain and likewise give peace of mind to the mother and increase nurse's confidence.
